# Emerging Technologies Supporting the Transition to a Circular Economy in the Plastic Materials Value Chain

**DOI:** 10.1007/s43615-022-00209-2

**Published:** 2022-09-01

**Authors:** Alejandro Aristi Capetillo, Fredric Bauer, Cristina Chaminade

**Affiliations:** 1 MSc in Innovation & Global Sustainable Development, Lund University, Lund, Sweden; 2 Environmental and Energy Systems Studies, Lund University, Lund, Sweden; 3 CIRCLE – Centre for Innovation Research, Lund University, Lund, Sweden; 4 Department of Economic History, Lund University, Lund, Sweden; 5 Department of Business and Management, Aalborg University, Aalborg, Denmark

**Keywords:** Circular economy, Emerging technologies, Plastics value chain, Sustainability transitions, Systematic literature review, ReSOLVE framework

## Abstract

Plastic waste has come to the forefront of academic and political debates as a global problem that demands an urgent solution. Promoted by policymakers, academia, and corporations alike, the circular economy model presents a viable path to reach more sustainable levels of development. Emerging and disruptive technologies can catalyse the transition to a circular economy, but their application to the transition of the plastic materials realm is not fully understood. Based on a systematic review of the literature, this paper aims to understand the role of key emerging technologies in the transition towards a circular economy in the plastic materials value chain, their potential impact, as well as the barriers of adoption and diffusion. Employing the ReSOLVE framework, the analysis reveals that rather than individual technologies, four technology sets associated with Industry 4.0, distributed economies, bio-based systems, and chemical recycling stand as major enablers of this transition. The complementarity of technologies and the change needed from a systemic perspective are discussed along with a proposal for governance and practical implementation pathway to overcome barriers and resistance to the transition.

## Introduction

There is no waste in nature. The resultant output from any given natural cycle works as an input to a complementary natural process. Through this principle, the whole planet is an interconnected complex and adaptive system [1]. In contrast, production and consumption systems in the modern economy follow a linear rationality in which resources are extracted, used, and then discarded at the end of their life. The unsustainability of this linear system has been the subject of academic discussion for decades [2–4]. The concept of a *circular economy* (CE) has gained momentum in the academic literature [5–7] as well as in the policy sphere [8–10] as an alternative to the predominant linear economic model. In short, the objectives of a CE are to design out waste and pollution, regenerate natural ecosystems, and significantly extend the useful life cycles of products and materials [8].

But shifting from linear globalised economies, focused on rapid production and distribution of goods at low costs, to circular economies that focus on a better use of resources and environmental regeneration, requires significant changes across most domains of society—in expectations, practices, regulations, and technologies. Such “long-term, multi-dimensional, and fundamental transformation processes through which established socio-technical systems shift to more sustainable modes of production and consumption” [11] rely on external pressures to open up for change and allow for the emergence of alternative practices and technologies. While no single technology could act as a silver bullet for a transition towards circular economies, there are emerging technologies that offer promising paths towards more circular modes of resource use and service delivery.

A growing body of literature is taking an interest in understanding the potential contributions of such emerging technologies to circular economies, as well as the limitations they have in doing so. The development of general digital technologies intended for industrial applications—commonly grouped together under the umbrella of Industry 4.0—has been identified as important enablers of many practices aligned with the aims of the circular economy [12–18]. But as the value and structure of different types of materials, products, and life-cycles differ widely, the opportunities of implementing emerging technologies are likely to differ across and throughout sectors and value chains.

Due to the durability, malleability, and tuneable properties—all available at low costs in global markets—it is hard to find any product that is not closely associated with plastics at some stage of the life cycle [16]. However, the mismanagement of this material has provoked numerous severe environmental problems on a global scale [17]. Plastic production continues to grow rapidly [18] and relies almost exclusively on fossil fuels—their manufacturing processes are highly energy-intensive and associated with large volumes of greenhouse gas emissions and other pollutants [19]. Packaging plastics, which constitute the single largest demand segment for plastics, have very short lifetimes—as do many other prominent uses of plastics, such as textiles used in the fast fashion industry [20]. While EcoDesign guidelines exist, they are still at an early stage and not applied at scale [21], causing plastic products to be commonly difficult to repair or dismantle since they were not designed to be recycled and so end up in landfills, incinerators, or discarded in natural environments. Plastics have permeated all domains of the world and can now be found literally everywhere, from the Arctic ice sheet [22] to human placentas [23], while estimates indicate that oceanic plastic materials will weigh more than fish by the year 2050 [24]. Thus, plastics are a key concern for the transition to a circular economy that aims to maintain the integrity of both ecosystems and global climate [25] while sustainably providing the necessary services and functions in the economy.

Hitherto, the research literature has not systematically analysed how the emerging technologies that have been identified as enablers of circular economy solutions interconnect with the dynamics of the plastic materials value chains. Furthermore, there is great demand for a better understanding of how innovative technologies can enable more sustainable solutions in value chains and how more circular alternatives to plastic waste management and recycling can be enabled [26, 27]. Understanding the opportunities, not only from the perspective of individual firms and business models in plastic value chains but also from a systemic perspective, is central to designing meaningful and effective forms of governance to shape and support the transition towards circular economies.

Intending to fill this gap in the literature, with this paper we aim to understand the role of key emerging technologies in the transition towards a circular economy in the plastic materials value chain, their potential impact, as well as the barriers of adoption and diffusion. We do so through a structured review of the relevant research literature.

The paper is organised as follows. The next section introduces the conceptual framework used in the paper, including a discussion of what is considered under the umbrella of emerging technologies and the plastic materials value chain. Section 3 details how the literature review was conducted. Section 4 showcases how different emerging technologies contribute to the circular economy action areas of Regenerate, Share, Optimise, Loop, Virtualise, and Exchange. Section 5 discusses the implementation challenges and barriers, as well as governance and proposed pathways to enable the transition. The final section concludes this writing and presents some reflections on the limitations of current research and future research venues.

## Conceptual Framework

### Circular Economy

With roots in disciplines like environmental economics, industrial ecology, and corporate sustainability (e.g. [4, 28]), the concept of CE is presently being promoted by policymakers, academia, and corporations as a viable path to enable sustainable ways of development [29] and accomplish the Sustainable Development Goals put forward by the United Nations [30]. While there are many definitions of the concept, each emphasizing a different aspect of it, we follow [6] who considers CE as.

“an economic system that is based on business models which replace the ‘end-of-life’ concept with reducing, alternatively reusing, recycling and recovering materials in production/distribution and consumption processes, thus operating at the micro-level (products, companies, consumers), meso-level (eco-industrial parks), and macro-level (city, region, nation and beyond), with the aim to accomplish sustainable development, which implies creating environmental quality, economic prosperity and social equity, to the benefit of current and future generations.” [6].

This comprehensive definition distinguishes circularity at different levels as well as across dimensions. In such a CE model, economic value is created by focusing on preserving the intrinsic value of products. Moreover, it recognises the importance of the economy in the current system of production and consumption by fostering efficiency and sufficiency at all scales [31]. Most importantly, the definition highlights that the goal of a CE is to not only lessen the harm associated with the linear economy but rather create a positive and reinforcing development cycle to sustain life in the long term [6]. The CE concept thus reflects three fundamental principles [31]: (1) preserve and enhance natural capital by controlling finite stocks and balancing renewable resource flows, (2) optimise resource yields by circulating products, components, and materials at the highest utility at all times in both technical and biological cycles, and (3) foster system effectiveness by revealing and designing out negative externalities.

The CE paradigm has also been subject to severe critiques regarding its applications in practice [32], its environmental constrains [7], and the limited attention to social sustainability [33]. In an analysis of the evolution of the concept since its inception, Reike et al. [32] highlight that, despite its great potential for resource value retention, most circular economy initiatives in practice have been focusing on the low-value retention aspects of recycling. In contrast, those that can potentially yield a higher impact in terms of resource efficiency, like remanufacturing, refurbishing, or repurposing, have been widely neglected by businesses and policymakers alike. Korhonen et al. [7] refer to the potential environmental limitations of the CE concept related to thermodynamic limits, its prospective contribution to global net sustainability, the risk of rebound effects, and path dependencies or lock-ins that prevent the adoption of more circular practices. Nonetheless, despite its critiques, the CE model continues to be considered a promising venue to contribute to the transition into a sustainable future.

Based on CE principles, the influential Ellen MacArthur Foundation has identified six action areas enabling such transition [34]: Regenerate, Share, Optimise, Loop, Virtualise, and Exchange. *Regenerate* refers to shifting to renewable energy and materials as well as reclaiming, retaining, and regenerating the health of ecosystems. *Share* aims at maximising the use of products by substituting individual ownership and by reusing them throughout the technical life through design and repair. *Optimise* refers to actions aimed to increase the efficiency in the production and the value chain. *Loop* aims at keeping products and materials in use in the economy for as long as is possible. *Virtualise* refers to substituting material products and services by digital ones, the best example being digital music and books substituting CDs and paper books. Finally, *exchange*, refers to actions aimed at substituting old materials with new, improved non-renewable materials.

Each of these action areas represent a business opportunity that, together with technological tools, habilitates companies and governments to create solutions and regulations that foster the shift towards a CE [34]. The framework, also known as the ReSOLVE framework, has largely been applied to the analysis of particular subsystems (mobility, energy) and sectors (textile) but its potential to analyse materials has not yet been fully explored. One such key group of materials is plastics. Its importance and impact will be discussed next.

### Plastic Materials Value Chains

The term ‘plastics’ encompasses a varied and still expanding group of polymers that are central in many industries, e.g. automotive, construction, packaging, textiles, and electronics. Introduced to mass markets in the mid-twentieth century, plastics that could feasibly be produced in cheap and massive quantities, meant that material production was no longer a practical constraint for the economy [35]. With time, what had been a niche innovation in an organic chemicals regime, successfully became a disruptive technology that would eventually transform the entire socio-technical system [36]. But also, as one of the main categories of the petrochemical industry, plastic’s steep uprising greatly contributed to the growth of the fossil-based economic system [37] and continues to do so today [18].

The plastic value chain is complex and touches upon several business sectors along its way. The majority of plastics are produced from fossil hydrocarbons, traditionally from naphtha—a by-product from refining crude oil into fuels—or from natural gas condensates such as propane and ethane [17]. These raw materials are then *cracked* to produce monomers like propylene and ethylene that are subsequently *polymerised,* yielding virgin polymers like polypropylene (PP) or polyethylene (PE). The virgin polymers, which commonly come in the shape of granulates (also called pellets or nurdles), are mixed with additional additives to obtain the desired properties for the intended application in a process called *compounding*. Subsequently, these plastics are *converted* into products through processes like moulding, blowing, or extrusion. The resultant items are either sold directly to the end consumers or used as components in more complex products [38].

**Fig. 1:**
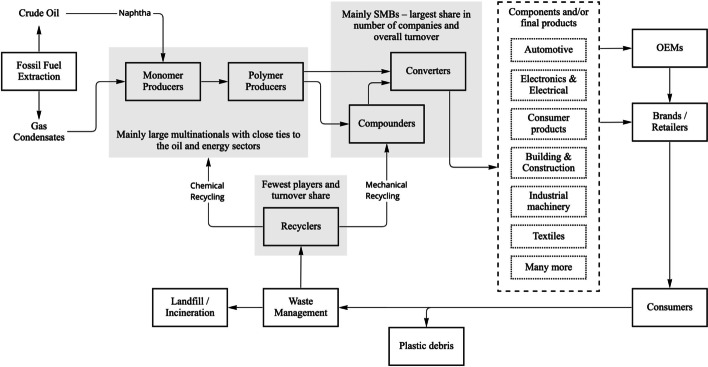
A representation of the plastic materials value chain. Source: Own diagram based on Nielsen and Bauer [38] and UNPRI [39]

After these final products are consumed or used, a large share becomes mismanaged waste (ending up in rivers or oceans) while a small portion of them is collected and sorted by waste management firms, who then pass the recyclable waste to the ‘recyclers’ or send the non-recyclable share to be either landfilled or incinerated. The recyclable portion is then processed to be used again, restarting at the polymer or conversion stages. A schematic image of plastic value chains is shown in Fig. 1.

Plastics are currently applied in a wide variety of products. A few examples include wrapping and caps (PP), shopping bags and general packing material (low-density PE), textiles (polyester, polyamide and acrylic—PP&A), bottles (high-density PE and polyethylene terephthalate—PET), mattresses and shoes (polyurethane—PU), disposable plates and cups (polystyrene—PS), and pipes (polyvinyl chloride—PVC). Given its wide use, it is not surprising that plastic materials value chains share many actors, institutions, and material elements with the fossil fuels and energy sectors as well as with other firmly established sectors such as the agro-food, electronics, transport, or textiles, among many others [40].

Due to the high heterogeneity of polymers, grades, and additives, the recycling of plastic is notoriously difficult [16, 41–43]. First, every polymer family (e.g. PET, PP, PVC, PS) has different physical properties; hence, different recycling techniques are needed to process them. Second, there is a massive and constantly growing quantity of plastic compounds that are designed without taking recyclability into consideration. Third, final products are rarely made of a single material and therefore, recycling processes, even if adequate for a single plastic type, might not work for products composed by several materials (e.g. wood, metal, etc.) or plastic types. And fourth, even if a plastic does manage to be recycled, it can be recycled so many times because, with every cycle, its properties degrade until a point when it cannot be recycled anymore. Consequently, understanding how the CE principles, through the usage of emerging technologies, can be applied in the plastic materials sphere and facilitate its transition, is important not only for the industry per se, but for the larger transformation of the contemporary production and consumption paradigm.

### Emerging Technologies in the Transition to a Circular Economy

The study of ‘emerging technologies’ has grown in the academic literature over the last years under different names such as Transformative Technologies (TT) [44], or Key Enabling Technologies (KET) [45]. Despite their different labels, the terms refer to a set of technologies that present specific characteristics [46]. (1) They are fundamentally different from what has previously been utilised to attain a similar goal, and so, they exhibit a *radical novelty*. (2) Compared to other technologies, they achieve a *relatively fast growth* rate. (3) They exercise a *prominent impact* on either a specific domain or a broader area within the socio-economic system by changing the constitution of actors, institutions or the interaction between them. (4) They are surrounded by *uncertainty and ambiguity* regarding their potential outcomes and applications, which could also result in undesirable or unintended consequences.

The literature on the impact of emerging technologies on the circular economy, although growing, is in its infancy. In a systematic literature review, Rosa et al. [47] indicate that most of the extant literature focus on the role of emerging technologies as enablers of the CE mainly through the widespread use of efficiency-driven digital technologies, including artificial intelligence (AI), blockchain, 3D printing, big data, or the internet of things (IoT). In fewer instances, emerging technologies are discussed in the framework of resource efficiency, remanufacturing, or product life cycle management, while studies focusing on supply change management and disassembly of products are scarce [47].

## Method

Considering the relative novelty of the circular economy and sustainability transitions concepts, as well as the inherently innovative aspect of the emerging technologies field, an exploratory approach is utilised in the present research. The paper employs a systematic literature review methodology put forward by Tranfield et al. [48] with the aim of “synthesizing research in a systematic, transparent, and reproducible manner”. We focused on the search, identification, appraisal, and synthesis of studies that combine two main concepts within the plastic materials value chain: circular economy and emerging technologies. As digital technologies are identified as central in the general literature on CE, it was decided to include this as a specific keyword in the search to capture its contribution even when not labelled as emerging or disruptive technology in the literature. Previous literature reviews discussed in “Conceptual Framework” section only partially overlap with the current paper approach since the data sources (additional journals included), industry focus (plastics), and analysis procedure (systematic review thematic) are fundamentally different.

For the initial scoping performed on April 3^rd^, 2021, a ‘briefsearch’ strategy is used. Further on, the ‘building blocks’ strategy is used for the construction and refinement of search queries using Boolean functions. Table 1 showcases the keyword clouds used for the search queries in the different databases. Appendix Table 3 details the search terms and queries employed for this step of the review.

**Table 1:** Keywords clouds for the search process

Topic	Circular economy	Plastic value chain		Emerging technologies	Sustainability transitions^a^
Subtopic	–	Value chain	Plastic	–	–
Keywords	circular economy, circular, circularity	supply chain*, value chain*, manufacturing, manufacturing chain*	plastic*, polymer*, monomer*, recycler*, plastic converter*	digital technolog*, emerging technolog*, disruptive technolog*	sustainability, sustainability transition*, transition*, sustainable, sustainable transition*, MLP, multi level perspective, regime*, socio-technical

In total, five databases were scanned for the systematic review of this paper: EBSCOHost, Emerald Insight, Scopus, Web of Science, and Wiley, comprising a comprehensive, high-quality, and cross-disciplinary review of published articles. A total of 502 unique papers were retrieved from the five databases, from which the most relevant ones were identified using the digital platform Covidence. References were imported to the platform, which allowed for the removal of duplicates, screening against title and abstract, full-text assessment, and based on other systematic reviews [13, 49], an additional step of including relevant cited papers (also referred to as ‘snowballing’) was also taken. A final set of 55 relevant papers were identified, all of which are published in peer-reviewed academic journals, written in English, and treat the interaction or exhibit a direct connection between emerging technologies, circular economy, and the plastics industry.

**Fig. 2:**
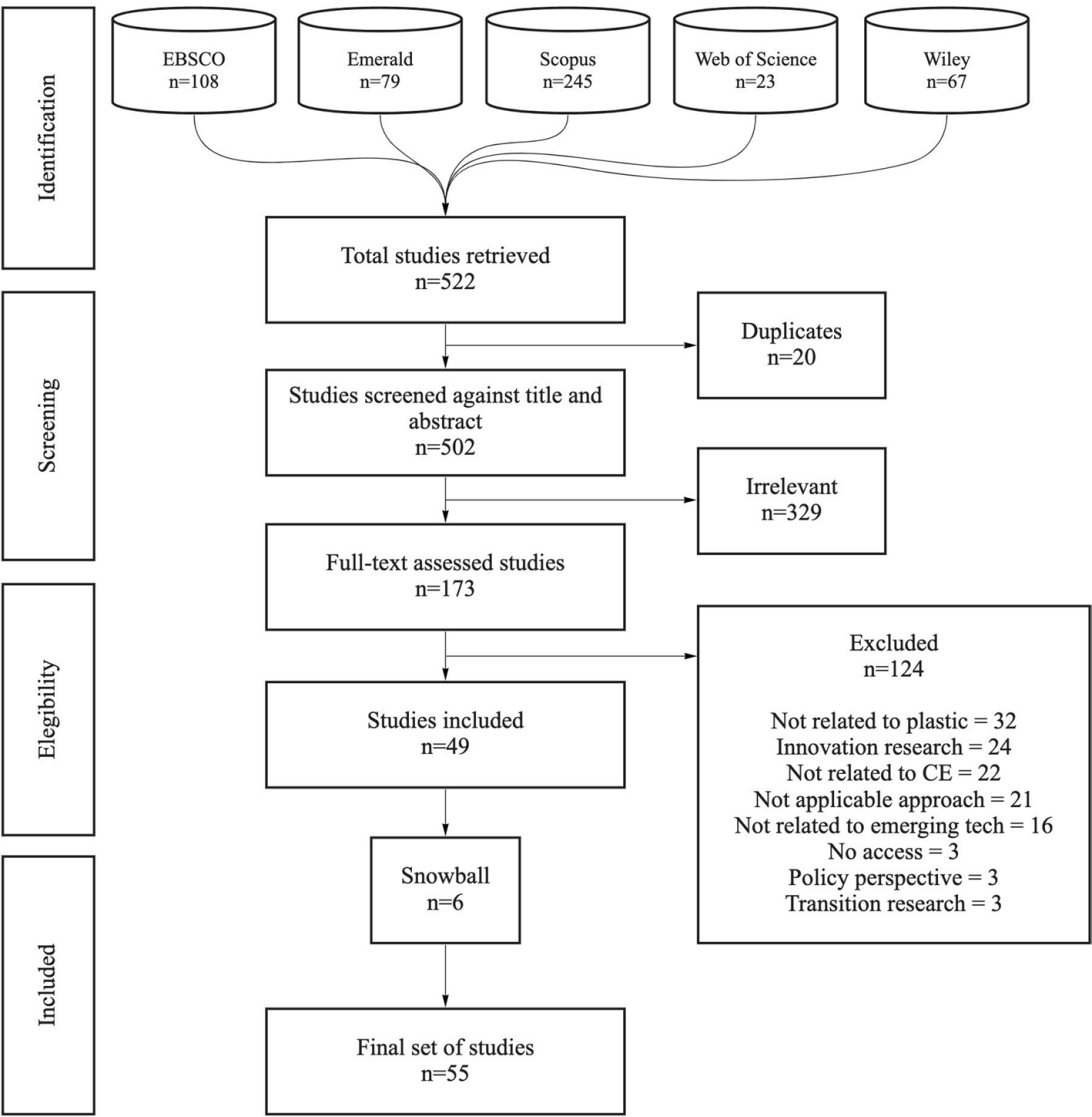
PRISMA diagram of the process of identifying relevant publications for the review

The next section discusses the main findings where two sets of analysis are presented. First, a descriptive analysis of the literature and second, a content analysis of the papers in terms of the emerging technologies discussed in the literature, its applications to different action areas in the transition to a circular economy, and the main barriers for their wider diffusion. Figure 2 presents the process and outcomes of each step in a PRISMA diagram. The final list of papers reviewed can be found in Appendix Table 4.

## Analysis

### Bibliometric Overview

**Fig. 3:**
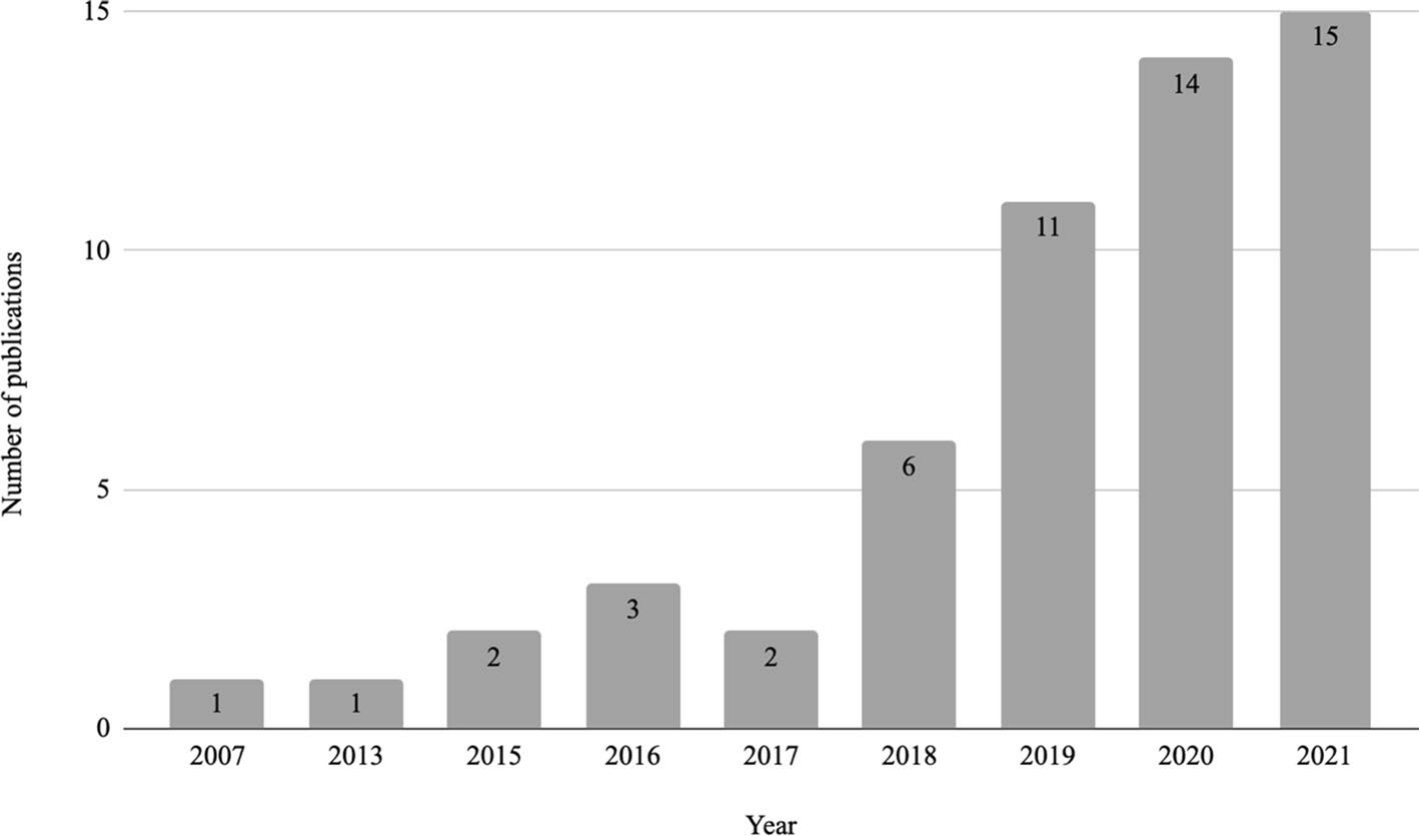
Publication year of the included studies (*N* = 55)

The descriptive analysis of the literature on circularity enabled by emerging technologies in the plastic field shows that it is a very recent phenomenon. As shown in Fig. 3 about 83% of the papers are published in the last three years, reflecting the novelty of the field.

When examining the authors’ geographic spread, around 38% of the articles involved collaboration between academics affiliated to institutions or research centres located in different countries. When looking at the articles published by researchers based in the same country, the UK and the USA represent the two largest sources of publications of the included studies accounting for 12% and 14%, respectively. Worth mentioning is the under-representation of authors affiliated to institutes in developing economies which account for only four studies of the total sample.

The study of the phenomenon in question is based on a wide range of disciplines that includes natural sciences (i.e. biology), physical sciences (i.e. chemistry), social sciences (i.e. business, economics), and information sciences (i.e. IT). Concepts such as ‘biorefineries’, ‘industrial symbiosis’, or ‘synthetic biology’ are examples of the cross-fertilisation process that these disciplines are going through. Relatedly, the articles reviewed for this research are published in a wide variety of journals. Totalling three, the *Journal of Cleaner Production* is the most significant contributor. Five other publications have two articles each. The remaining journals have only one included article each.

### Emerging Technologies in the Manufacturing Plastic Value Chain

A total of 15 emerging technologies related to the transition towards circularity in the plastic materials value chain were identified in the reviewed publications, as shown in Table 2. Biopolymers and biorefineries are the emerging technologies which have received most attention in the literature, followed by digital technologies, including artificial intelligence, blockchain and internet of things.

**Table 2:** Summary of the emerging technologies used in different circularity action areas by number of mentions, technology set, and CE action area

Technologies	Number of mentions in the literature	Technology set*	Action area								
		***I4.0***	***DE***	***Bio***	***CR***	***Regenerate***	***Share***	***Optimise***	***Loop***	***Virtualise***	***Exchange***
Biopolymers	24			X		X	X	X	X		
Biorefineries	23			X					X		
Internet of Things (IoT)	16	X	X					X	X		
Artificial Intelligence (AI)	14	X	X					X	X		
Blockchain	14	X	X			X	X		X	X	X
Synthetic Biology	12			X		X		X	X		
Nanotechnologies	11	X						X	X		X
3D Printing	10	X	X					X		X	X
Robotics	10	X							X		
Chemical Recycling	10				X	X			X		
Big Data	9	X	X					X		X	
Cloud Computing	7	X	X					X			
Augmented and Virtual Reality	5	X								X	
Process Intensification	4	X		X				X			
Microrecycling	3		X						X		

* **I4.0**: Industry 4.0; **DE**: Distributed economies; **Bio**: Bio-based systems; **CR**: Chemical recycling

**Fig. 4:**
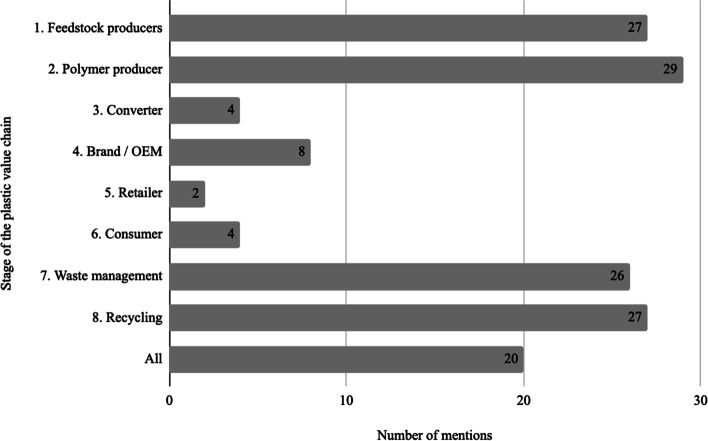
Stages of the plastic materials value chain impacted by the emerging technologies identified in the included articles

Considering the individual technologies, the first interesting finding relates to the **stages** of the value chain that these emerging technologies are relevant for. As can be observed in Fig. 4, the largest group addresses the early stages of the value chain, feedstock and polymer producers, while the second largest group addresses the end-of-life stages of waste management and recycling. Very few specifically address the mid stages of plastic value chains. Interestingly, a final group of 20 papers present technologies that impact all the stages in the value chain and could therefore indicate a possible system-level change.

Beyond individual technologies, what the literature highlights is the complementarities between them. Among these emerging technologies, we identify **four technology sets** that have the potential to disrupt the current plastic materials regime in four different ways: (1) increasing efficiency and automation capabilities (Industry 4.0), (2) enabling a shift in the production-consumption system (distributed economies), (3) facilitating the development of high-added value products from biological materials (bio-based systems), and (4) reducing the need for raw materials to produce high-quality recycled plastic (chemical recycling).

First, according to the literature, the data exchange and automation capabilities enabled by **Industry 4.0** technologies exhibit great potential for increased circularity in the manufacturing stages of the plastic value chain. Favourable forces behind this group of technologies include a promise of efficiency and productivity increase [12–14, 26, 44, 50–57], the generation of positive marketing messages towards the consumers [14, 44, 58, 59], an enabling of materials’ re-utilisation [13, 50, 52–54, 60, 61], a potential seizing of mixed waste sources [27], the enabling of transparency and collaboration among actors [26, 50, 51, 56, 58–60], and a prominent societal impact which aligns to current political discussions [27, 62].

The concept of **distributed economies** refers to shifting the economic paradigm into more local, or even personal systems of sourcing, manufacturing, consumption, and recycling [63]. The combination of emerging technologies that enable this novel concept are 3D printing, IoT, blockchain, AI, and cloud computing as well as small-scale chemical transformation processes enabling shifts in the production-consumption and socio-economic systems rather than to the increase in efficiency and automation capabilities featured in the previous category. Examples of these solutions include ‘microrecycling’ [64], ‘peer-to-peer circularity’ [62], and the 3D printing-enabled production/consumption socio-technical system [43, 65, 66].

Supporting arguments for the adoption of this set of technologies in the plastic manufacturing value chain include a complete redefinition of the concepts of ‘waste’ and ‘value’ [64, 66], the re-utilisation of materials [43, 62, 64], the employment of mixed waste streams through a decentralised form of addressing current issues with the sorting and collection of waste [64, 66], and a promise of society-wide impact [43, 64, 65]. Even more, by enabling auto-sufficiency and enclosing the production of goods into a smaller scale and geography, activities that are currently perceived as non-profitable may become so [58, 64, 66].

The third technology set, **bio-based systems,** expands on the idea of ‘fabricating value-added products from materials of biological origin’ and includes the set of technologies (e.g. synthetic biology), inputs (e.g. biowaste), processes (e.g. anaerobic digestion), and products (e.g. biopolymers) entailed in the concepts of biorefineries and bio-based materials. Leveraging on the benefits of a local or regional production and the ‘economies of scope’ model, the bio-based systems paradigm set presents a viable alternative to the systemic reliance on fossil fuels [67–70]. The literature argues that bio-based systems enable material’s re-utilisation and fundamentally change the perception of ‘waste’ and ‘value’ [61, 70–80]. They also use a mixed and/or contaminated waste source (although not uniquely fossil-based plastic) [41, 70, 74, 75, 77, 78, 81, 82], keep efficiency and productivity as a priority by focusing on the manufacturing of several products [68, 70, 72, 74–79, 81, 83, 84], and are notably aligned to the political discussion topics through the concept of ‘bioeconomy’ [67, 68, 75, 85].

Finally a fourth set of technologies revolves around the concept of **chemical recycling.** It refers to processes chemically modifying plastics to yield a high-quality recycled plastic material. Most chemical recycling technologies aim to break the polymers’ chemical bonds, converting them to monomers that can be processed again just as if coming from a virgin source [86]. Several chemical recycling techniques like pyrolysis, solvolysis, gasification, and dissolution/precipitation further enhanced by chemical procedures such as microwave heating, plasma reactors, or the usage of compound chemicals and supercritical fluids can be particularly helpful for the chemical recycling of plastic [57, 62, 86, 87]. Arguments in favour of this set of technologies include the re-utilisation of materials and redefinition of waste [53, 86–89], an industry-wide impact promise [57], and an alignment to current political discussions [62]. Most importantly, chemical recycling technologies align with the current corporate dynamics in terms of installed capacity for both the production of recycled plastic and, increasingly, the utilisation of mixed and contaminated plastic waste [86, 89].

### Relevance of Emerging Technologies for CE Action Areas

The articles were analysed using a thematic lens to identify the emerging technologies that are explicitly mentioned in relation to a particular action area of the circular economy, namely *Regenerate, Share, Optimise, Loop, Virtualise*, and *Exchange*.

Table 2 plots how the different emerging technologies are currently discussed in relation to the CE action areas in the plastic materials industry. It becomes clear that most of the focus hitherto has been on how emerging technologies can contribute to closing different *loops* and *optimising* the use of plastic throughout the value chain. On the other hand, even though the *regenerate* and *exchange* action areas are not quantitatively associated with many technologies, they relate to some of the most potentially disrupting ones at the systemic scale and from a standalone perspective (*blockchain, 3D printing, nanotechnologies, synthetic biology, chemical recycling, and biopolymers*). Less emphasis has been given to the impact of new technologies on the *share* and *virtualise* action areas.

#### Regenerate

The ‘Regenerate’ action area relates to the restoration of the Earth’s natural cycles and ecosystems. It includes a transition to renewable energy, materials, and a renewal of ecosystems’ health. *Bio-based polymers*, *synthetic biology*, the importance of system-wide changes, and technologies aimed to restore the balance of marine ecosystems are associated with this CE action area.

The concept of ‘bioeconomy’ [67, 68, 81] relates to the “production of renewable biological resources and the conversion of these resources and waste streams into value-added products, such as food, feed, bio-based products, and bioenergy.”[90].^1^ When it comes to the plastic materials industry, the technologies behind the production of bio-based polymers such as PLA, PCL, or PHA are currently being tested, scaled, and are increasingly providing an economically and environmentally viable platform for the substitution of fossil-based plastics [91]. Different types of bio-based and biodegradable polymers currently available include synthetic, microbial, and natural biopolymers [69]. Examples of applications include the use of agricultural nets made out of biopolymers to increase crop yields while minimising the use of direct contact pesticides and plastic waste [41].

Since biopolymers are fabricated from organic feedstock or biomass, a merge between chemistry and biology is evident. The *synthetic biology* field enables the use of photosynthesis to capture solar energy and generate building blocks for materials in the bioeconomy [83]. Gene editing of crops and plants to confer desired characteristics during the harvest (e.g. pesticide-free crops [84]), production processes (e.g. biocatalysts [71]), and final products (e.g. anti-fungal properties) pose as critical paths to overcome future large-scale production challenges.

However, aside from the high-technology development perspective, system-wide work needs to be done for the bio-based and renewable materials industry to properly expand. Namely, securing a sustainable feedstock availability at the regional level, fostering collaboration between supply chain actors, and increasing the acceptance of biotechnologies [67]. Additionally, an improvement of agricultural performance and a more efficient use of biomass where the circular economy concept is included must be achieved [68]. Furthermore, a shift towards renewable energy sources to be used in the manufacturing processes also plays a key role as it lowers the final product carbon footprint (e.g. in food products [91]) and thus, increases its customer appeal.

#### Share

The ‘Share’ action area focuses on the reuse and sharing of assets and products as well as extending the overall product’s life [34]. Technologies empowering the inner loops in the circular economy model and the shift in business models stand out as the most transformative.

*Blockchain* is the most mentioned emerging technology due to its capabilities to trace assets or products along the sharing/consumption journey [51, 52, 62], and for its security, recordkeeping, and immutability of information features [50]. Additionally, several papers discuss the physical location tracking benefits that *IoT* technologies provide [13, 50, 60]. The mix of these two technologies (*blockchain* and *IoT*) facilitate the sharing and re-usage of objects by providing a trustworthy physical and digital tracking medium. For example, companies could be able to share or rent construction equipment based on a project’s needs and be sure about the location, usage history, and need for maintenance without the need of human intervention.

The increasing amounts of plastic types used in the manufacturing of electrical and electronic devices, as well as the incompatibility between the individual classes of polymers used, are presented as barriers for recyclability that could be minimised through a design-for-sustainability approach [15, 53, 92]. However, the number of articles exploring the set of technologies that could support the *maintenance, design, and durability* of plastic materials is still very limited.

#### Optimise

The ‘Optimise’ action area concentrates on increasing efficiency either through performance improvement, waste reduction, or leverage of novel technologies [34]. *Synthetic biology*, *Process Intensification*, and *Industry 4.0 technologies* display pivotal transformational qualities.

A common strategy to increase performance levels is to develop ad-hoc polymers based on specific performance needs. However, this approach makes the end products more difficult to recycle and defeats the purpose of using bio-based materials. *Synthetic biology* techniques focused on improving biopolymer’s properties, rather than developing new compounds, showcase great potential—food packaging with antimicrobial qualities [93] and enhanced physical properties [73], or an extension of shelf life of fruits and vegetables by using biopolymer-based nanocomposites [68, 72] are becoming increasingly viable options.

Either in combination or from a standalone perspective, different authors highlight several emerging technologies that reduce waste [44], enable the closure of resource flows, and create value while reducing costs and increasing revenues [54]. A combination of technologies that could mean a considerable leap forward in terms of efficiency in the production and supply chain processes is made up of *Big Data*, *AI* through its various branches (e.g. machine learning, computer vision, automation capabilities), and *IoT* [13, 14]. Seen from a high-level perspective: *AI* provides the logic and the processing of data that is either supplied in real-time by *IoT* sensors or based on the historical performance (*Big Data*). This mix becomes even more interesting when adding *autonomous robots* as it extends the capabilities of AI by giving it control over manufacturing devices, enabling continuous monitoring and optimisation of performance and processes [12, 55].

The efficiency-driven *Process Intensification (PI)* model employs chemical engineering and process optimisation techniques to accomplish a cleaner and more efficient use of manufacturing resources [91]. *PI* improves resource efficiency and reduces waste by “maximizing mass, heat, and momentum transfer” throughout production stages [94]. The impact of *PI* can be amplified by combining it with other emerging technologies such as *additive manufacturing* to “print” custom-made parts that concretise a manufacturing plant’s layouts [12], with *AI* technologies for real-time process optimisation and decision-making [94], or with organic synthesis microreactors that shift production processes from batch to continuous while delivering higher efficiency in the production of biofuels [71, 94].

A third technological blend is constituted by *Big Data Analysis* and *Cloud Computing* where the large-scale processing of historical datasets would enable more accurate forecasts from both the supply and demand sides of diverse economic sectors, such as fashion [55] and refined chemicals [54]. This translates into a better seizing of resources and thus, less waste.

Lastly, when looking at these technologies independently, using *IoT* technologies for unique items tracking [13, 60] or for an improved management of e-waste and agricultural waste [27] stand out. Furthermore, *IoT*-enabled data collection from waste flows would help organisations capture an incremental value from the tightening of resource flows via cost savings [54].

#### Loop

The ‘Loop’ action area entails the necessary processes and technologies to reintroduce materials back into the system either through remanufacturing, recycling, or extracting valuable matter from waste [34]. Technologies that enable the *closing of loops* such as *blockchain*, *chemical recycling*, *microrecycling*, *biopolymers*, and *biorefineries* are highlighted next.

The remanufacturing of products or components is an important inner loop within the CE model since it extends the life of components and thus lowers the associated manufacturing emissions along the product’s life cycle. Considering the durability and composition specificity of the plastic materials used in certain industries (e.g. automotive and electronics), one would think that remanufacturing processes play an essential role; however, this is not the case. The use of *Radio Frequency Identification* (RFID) tags that track products and material flows to enable value recovery through reusing, repairing, and remanufacturing is the only identified technology aligned to this sub-area [13]. The main reason behind this apparent lack of technological focus in the remanufacturing loop relates to a lack of infrastructure that enables ‘reverse logistics’ processes to gather products amid the End-of-Life stage either in the form of mono-plastic waste stream [53] (e.g. only PVC plastics) or from a general plastic waste perspective [44, 86].

*Recycling materials* is not a new concept, but it is still prominent in transitioning to a CE model in the plastic materials value chain. Again, *Blockchain* technology is highly relevant in this topic due to its two main functionalities: transparency/traceability and security/reliability/immutability [15, 50]. On the one hand, the transparency and inherent traceability of materials along the entire value chain provide the needed visibility of an end-product’s material composition and allows the recyclers to know whether and how a product should be recycled [61]. Knowing, for example, the polymer composition, provenance, or the number of times a given plastic packaging [53] or garment [59] has been recycled, optimises the corresponding recycling processes. On the other hand, the security, reliability, and data immutability of a decentralised network allows for greater degrees of trust between the different entities involved in the value chain [52]. This, in turn, results in better communication and collaboration that not only lower the operational issues of recycling [70] but also improve the transport and logistic systems throughout a product’s delivery stages [95].

*Microrecycling* is a disruptive concept that aims to tackle the main issues of recycling waste from electrical and electronical equipment (WEEE), but one that can also be applied to other plastic value chains [64]. The central idea of microrecycling is to use a distributive recycling approach to avoid the technical and financial barriers faced by the processes and companies involved in the scaling of material recycling. Thus, instead of having a centralised waste management system, the processing and reintroduction of valuable materials into the system happens at a smaller scale through ‘microfactories’—providing new life to previously difficult-to-process waste while producing added-value materials at a local level.

Several articles emphasise the role that *biorefineries* could exert on the plastic materials value chain [68, 72, 74–78, 81, 82, 96]. A *biorefinery* is a processing facility that utilises several technologies and equipment to convert biomass into products such as fuel, chemicals, energy, and other materials [81]. It is the “renewable equivalent of a fossil-based (petroleum) refinery” [97]. Considering that one of the main outputs of *biorefineries* are *biopolymers*, the effect of this concept cannot be overstated. In essence, it signifies the end of the over-dependence on fossil fuels to fabricate this ubiquitous material and a huge step towards a bio-based, closed-loop economy. The fact that *biorefineries* are designed to process and deliver various products from diverse waste streams in a sustainable manner is essential to support the economic viability of the *biorefinery model* [74, 78, 86]. Lastly, a key aspect of the three processing steps is that the use of enzymes and other genetically-modified organisms, also previously referred to as *synthetic biology*, is the rule rather than the exception.

#### Virtualise

The ‘Virtualise’ action area relates to the direct or indirect substitution of resources by delivering utility virtually [34]. Although a crucial action area on other grounds, in the case of plastic material, this is the action area that has less impact.

The available articles on the topic focus on the use of *blockchain* to virtualise and automate contracts [50] and the use of *Augmented Reality (AR)* or *Virtual Reality (VR)* technologies to simulate a real-life production facility or process before building/implementing it [55], which might be of use in designing products for maintenance, durability, and upgradability.

#### Exchange

The ‘Exchange’ action area comprehends the shift towards replacing legacy ways of production and consumption by using more advanced non-renewable materials, the application of new technologies, and the choice of new products or services [34]. *Nanotechnologies* together with *3D printing* machines and systems deliver promising transformational avenues.

The use of *nanotechnologies* to improve the performance of plastic materials [69, 73] or the enhancing of concrete by combining it with non-recyclable plastic [89] showcase how new techniques and materials can improve legacy systems.

A potentially disruptive impact that the mainstream adoption of *3D printing* technologies by consumers and industries is envisioned by several researchers [43, 65, 66]. The authors envision virtualisation of the entire plastic supply chain, from transport logistics to production and retail, with the increased adoption and advancement of this technology by “closing the loop at a local level of scale by matching local waste sources with demand from 3D printing” [66]. In summary, 3D printers will enable final consumers to ‘print’ their own goods, based on their own specifications, using their own waste (either plastic, metal, or even biowaste) [66], and only rely on ‘product design’ providers who will sell and virtually deliver the software needed for the 3D printers to personally manufacture the product [65]. For more complex items, a ‘coproduction’ model consisting of distributed and locally-framed supply chains, is presented as a more efficient and environmentally conscious alternative to the current globally-entangled supply chain systems [65]. Lastly, 3D printing is visualised as an enabler of the shift into a socio-technical system focusing on mono-material, value-cycling, and autonomous dynamics that prioritise function over form and scope over scale [43, 64].

Nevertheless, several obstacles limit the adoption of *3D printing* in the plastic value chain. The systemic resistance that originates from an organisational culture based on profitability and risk-avoidance [43, 65, 66], the current quality of 3D-printed products, and market acceptance of products coming from recycled materials [66] are mentioned as important barriers.

### Implementation Challenges and Barriers

The literature suggests there is a great potential for the new technologies to enable the transition towards circular economies in the plastic value chain. However, their adoption is limited by significant barriers that relate to (1) industrial lock-ins, (2) misalignment with current corporate logics (3) production systems dynamics, (4) the maturity stage of some of the technologies with its associated growth-related concerns and economic trade-offs, and (5) lack of understanding of the technology or its effects. Public policies can play a significant role in lowering some of the barriers and thus facilitating the transition as will be discussed next.

In terms of industrial lock-ins, the plastic value chain is mature and well established. Hence, it is expected for the current actors, with massive locked-in investments, to defend their position and technologies in place [41, 43, 62, 66, 75, 83, 89, 96, 98]. Financially, the costs of changing the installed capacity in terms of machinery and processes are elevated, particularly considering the high risk and high uncertainty associated to the new technologies. Industrial lock-ins are well known in the literature and the particularities of lock-ins in the plastics sector have also been studied [17, 18].

Secondly, risk aversion is a defining characteristic of the current corporate landscape that permeates the pace, direction, and magnitude of the transition towards the adoption of circularity-enabling disruptive technologies. Even if a specific technology brings clear benefits in terms of efficiency or quality, an industry-wide consensus and a shared understanding of the benefits is necessary to propel the adoption of innovative technologies [44, 56, 57, 67, 70, 75, 77, 94, 95]. Sometimes, even if big actors are testing and pushing towards the spread of new tools that enable circularity, smaller players keep being sceptical and wait until it becomes a requirement from the market or from the regulatory side to embrace novel technologies [26, 44, 50, 62, 70, 83, 91, 92].

Thirdly, the current production-consumption paradigm is focused on the valorisation of single products and so, it is wasteful [44, 62, 75, 75, 77–79, 84, 88]. Focusing on other possible production outputs, as well as building a secondary raw materials market are presented as feasible alternatives that have a potential to grow with the aid of the emerging technologies described [15, 53, 57, 64]. However, some of the potential bio-based substitutes for plastic are still in their infancy [77]. While large efforts have been made on investigating the potential of biorefineries [70, 74–76, 78], much research needs to be done on how to transform the new inputs into products and materials in order to ensure a sustainable, high-quality, and continuous feedstock supply [67, 75, 76]. Furthermore, the current consumption system is largely based on the single use of plastic products, alluding to a significant potential for reusing and sharing while also indicating that the barrier is not only on the producer side but also from the consumer behaviour perspective [44].

Fourthly, the exponential growth of the fossil-based plastic system during the past decades also meant that companies became extremely efficient in both quality and costs along the entire production process of plastics. Therefore, it is very difficult for any novel solution to compete with the existing players merely on economic grounds [76–78, 81]. For example, virgin plastic packaging is the best available option for food items regarding both material properties and cost [44, 69, 93], and while several other alternatives for packaging exist, their elevated production costs become prohibitive to be used on a large scale [73]. Even more, the comparatively low productivity/efficiency, as well as the need for massive investments in R&D and machinery that often come without a clear positive environmental outcome, also stand in the way of the growth and diffusion of the reviewed technologies [27, 41, 44, 50, 55, 62, 68, 72, 76, 81, 82, 89, 91, 96].

Fifth, the analysis reveals a lack of technical and conceptual understanding of the technologies and their effects among many actors. There is also a shortage of skills and organisational learning capabilities to absorb and implement these innovative technologies [41, 84, 86], in particular among small and medium-sized companies. This could be due to the lack of information, but also to the low technical maturity and the complementary nature of many of these technologies, which makes the outcomes even more uncertain.

What the foregoing discussion highlights is that the adoption of emerging technologies for circular economy in plastic material value chains must co-evolve with social and institutional innovations [50, 56, 59, 66, 68, 75, 91, 98, 99]. That is, reconfigurations of actors, networks, policy frameworks, and value chains as well as a new set of incentives to phase out old technologies and foster the growth of cleaner technological solutions [14, 43, 59, 62, 67, 68, 70, 78, 80, 83, 86, 91].

## Conclusions

With data obtained through a systematic literature review of 55 academic articles, this paper seeks to investigate how emerging technologies are seen to potentially contribute to the transition to a circular economy in plastic material value chains.

What the literature review highlights is the complementarity between different technologies. The review has identified that, rather than individual technologies, there are four sets of technologies that have the potential to enable increased circularity in the plastic materials value chains: Industry 4.0, distributed economies, bio-based systems, and chemical recycling technologies. From different angles and varying scope, each of these technology sets can operationalise the circular economy principles and represent a fundamental shift in the current modus operandi of the socio-technical regime in question across the identified action areas Regenerate, Share, Optimise, Loop, Virtualise, and Exchange.

While none of the identified technology sets delivers a fully circular solution, technologies that contribute to an *optimised* use of plastic materials by increasing efficiency and productivity along all steps of the value chain offer promising transformation pathways. This is clearly the case of Industry 4.0. The primary mechanisms for these technologies to enhance circularity are those that enable data and information exchange between stakeholders, as well as process automation at the manufacturing, consumption, and recycling stages.

Moreover, in the transition towards a CE in the plastic materials realm, an important role is taken by emerging technologies aimed at reintroducing materials back into the system or ‘*closing the loop*’. These technologies, which result from a merge between several knowledge fields (e.g. chemical engineering, industrial biotechnology), can enable the production, upgrading, and (re)processing of new and existing types of polymers to be better suited for circular life cycles.

Although technologies encompassing the *regeneration* of natural ecosystems, the *sharing* of assets, and the *exchange* of legacy ways of production and consumption are not, by comparison, quantitatively associated with many technologies, they relate to some of the most potentially disruptive at the systemic level. This highlights the multi-level and system-wide shift that is needed for these, and their burgeoning industries, to thrive.

As with any socio-technical system, the development, adoption, and expansion of emerging technologies largely depend on the way humans interact with them. On the one hand, this means that these tools are also likely to be deployed and used in ways that do not essentially contribute to circularity, so it is crucial to monitor the development of these technologies closely to ensure that their circular potential is properly materialised. On the other hand, it means that they are subject to face adoption and expansion obstacles not uniquely related to the technological development nature, but also from a cognitive, perceptual, organisational, market, and systemic perspective.

Literature review papers are limited by the content and availability of published information. The fact that a particular technology or action area is discussed to a varying extent in the literature might underscore its importance in the real world. Research into the enablers and barriers of adopting novel technologies for circularity in the plastic materials domain should be complemented with data collected from practitioners. Moreover, as previously noted, a socio-technical transition as the one envisaged here will require profound transformations in both the technical and social systems. While the focus of this paper has been on the technologies, most of the barriers to their adoption are related to non-technological aspects. Future research should investigate the co-evolution of technological and non-technological innovations in the transition to a circular economy in the plastic materials value chain.

## Appendix 1

Table 3

**Table 3:** Databases and Search Queries

Database	Query string and Expanders/Limiters
EBSCO Host	**Databases:** Academic Search Complete, Business Source Complete, EconLit, GreenFILE, Library, Information Science & Technology Abstracts with Full Text
	**Expanders:** Also search within the full text of the articles, Apply equivalent subjects**, Limiters:** Scholarly (Peer Reviewed) Journals
	**Query string:** ( ("sustainability" OR "sustainability transition*" OR "sustainable transition*" OR "MLP" OR "multi level perspective" OR "regime*" OR "socio-technical")) AND ( ("supply chain*" OR "value chain*" OR "manufacturing" OR "manufacturing chain*")) AND ( ("plastic*" OR "polymer*" OR "monomer*" OR "recycler*" OR "plastic converter*")) AND ( ("digital technolog*" OR "emerging technolog*" OR "disruptive technolog*")) AND ( ("circular economy" OR "circularity"))
Emerald Insight	**Query string:** (content-type:article OR content-type:"case study" OR content-type:"earlycite article") AND (( "sustainability" OR "sustainability transition*" OR "transition*" OR "sustainable" OR "sustainable transition*" OR "circularity" OR "circular economy" OR "circular") AND ( "MLP" OR "multi level perspective" OR "regime*" OR "socio-technical") AND ( "supply chain*" OR "value chain*" OR "manufacturing" OR "manufacturing chain*") AND ( "plastic*" OR "polymer*" OR "monomer*" OR "recycler*" OR "plastic converter*") AND ( "digital technolog*" OR "emerging technolog*" OR "disruptive technolog*"))
Scopus	**Query string:** ( "sustainability" OR "sustainability transition*" OR "transition*" OR "sustainable" OR "sustainable transition*" OR "circularity" OR "circular economy" OR "circular") AND ( "MLP" OR "multi level perspective" OR "regime*" OR "socio-technical") AND ( "supply chain*" OR "value chain*" OR "manufacturing" OR "manufacturing chain*") AND ( "plastic*" OR "polymer*" OR "monomer*" OR "recycler*" OR "plastic converter*") AND ( "digital technolog*" OR "emerging technolog*" OR "disruptive technolog*") AND ( LIMIT-TO ( DOCTYPE, "re") OR LIMIT-TO ( DOCTYPE, "ar")) AND ( LIMIT-TO ( LANGUAGE, "English")) AND ( LIMIT-TO ( SRCTYPE, "j"))
Web of Science	**Databases:** WOS, BIOSIS, CABI, FSTA, KJD, MEDLINE, RSCI, SCIELO, ZOOREC
	**Period:** Auto, **Language:** Auto
	**Query string:** TS = ( "sustainability" OR "sustainability transition*" OR "transition*" OR "sustainable" OR "sustainable transition*" OR "circularity" OR "circular economy" OR "circular" OR "MLP" OR "multi level perspective" OR "regime*" OR "socio-technical") AND TS = ( "supply chain*" OR "value chain*" OR "manufacturing" OR "manufacturing chain*") AND TS = ( "plastic*" OR "polymer*" OR "monomer*" OR "recycler*" OR "plastic converter*") AND TS = ( "digital technolog*" OR "emerging technolog*" OR "disruptive technolog*")
Wiley	**Applied filters:** Journals
	**Query string:** "sustainability transition" OR "circular economy" OR "sustainability transitions" OR "multi level perspective" OR "MLP" "digital technology" OR "emerging technology" OR "disruptive technology" OR "digital technologies" OR "emerging technologies" OR "disruptive technologies" “plastic” OR “plastics” "supply chain" OR "value chain"

## Appendix 2

Table 4

**Table 4:** Articles included in the literature review

#	Authors	Year	Title	Publication
1	Acioli, Scavarda and Reis	2021	Applying Industry 4.0 technologies in the COVID-19 sustainable chains	International Journal of Productivity and Performance Management
2	Andrae et al	2016	Practical Eco-Design and Eco-Innovation of Consumer Electronics–the Case of Mobile Phones	Challenges
3	Arun et al	2020	Remodeling agro-industrial and food wastes into value-added bioactives and biopolymers	Industrial Crops & Products
4	Bag et al	2018	Industry 4.0 and supply chain sustainability: framework and future research directions	Benchmarking: An International Journal
5	Bag et al	2021	Barriers to adoption of blockchain technology in green supply chain management	Journal of Global Operations and Strategic Sourcing
6	Basumatary et al	2020	Biopolymer-based nanocomposite films and coatings: recent advances in shelf-life improvement of fruits and vegetables	Critical Reviews in Food Science and Nutrition
7	Bauwens, Hekkert and Kirchherr	2020	Circular futures: What Will They Look Like?	Ecological Economics
8	Bezama et al	2019	Resources, collaborators, and neighbors: The three-pronged challenge in the implementation of bioeconomy regions	Sustainability
9	Birtchnell and Urry	2013	Fabricating Futures and the Movement of Objects	Mobilities
10	Böckel, Nuzum and Weissbrod	2021	Blockchain for the Circular Economy: Analysis of the Research-Practice Gap	Sustainable Production and Consumption
11	Boffito and Fernandez Rivas	2020	Process intensification connects scales and disciplines towards sustainability	The Canadian Journal of Chemical Engineering
12	Bongomin et al	2020	Industry 4.0 Disruption and Its Neologisms in Major Industrial Sectors: A State of the Art	Journal of Engineering
13	Braglia et al	2021	Managerial and Industry 4.0 solutions for fashion supply chains	Journal of Fashion Marketing and Management: An International Journal
14	Clark, Trimingham and Storer	2019	Understanding the views of the UK food packaging supply chain in order to support a move to circular economy systems	Packaging Technology and Science
15	Clarke	2019	Synthetic biology—pathways to commercialisation	Engineering Biology
16	Dalrymple et al	2007	An integrated approach to electronic waste (WEEE) recycling	Circuit World
17	Dijkstra, van Beukering and Brouwer	2021	In the business of dirty oceans: Overview of startups and entrepreneurs managing marine plastic	Marine Pollution Bulletin
18	Erickson et al	2021	End-to-end collaboration to transform biopharmaceutical development and manufacturing	Biotechnology and Bioengineering
19	Eseyin, Steele and Pittman	2015	Current trends in the production and applications of torrefied wood/biomass—A review	Bioresources
20	Esmaeilian et al	2020	Blockchain for the future of sustainable supply chain management in Industry 4.0	Resources, Conservation & Recycling
21	Fermoso et al	2018	Valuable Compound Extraction, Anaerobic Digestion, and Composting: A Leading Biorefinery Approach for Agricultural Wastes	Journal of Agricultural and Food Chemistry
22	Fierascu et al	2019	Recovery of Natural Antioxidants from Agro-Industrial Side Streams through Advanced Extraction Techniques	Molecules
23	Garmulewicz et al	2018	Disruptive Technology as an Enabler of the Circular Economy: What Potential Does 3D Printing Hold?	California Management Review
24	Gligoric et al	2019	SmartTags: IoT Product Passport for Circular Economy Based on Printed Sensors and Unique Item-Level Identifiers	Sensors
25	Gontard et al	2018	A research challenge vision regarding management of agricultural waste in a circular bio-based economy	Critical Reviews in Environmental Science and Technology
26	Howson	2020	Building trust and equity in marine conservation and fisheries supply chain management with blockchain	Marine Policy
27	Hussain, Mishra and Vanacore	2020	Waste to energy and circular economy: the case of anaerobic digestion	Proceedings of the Estonian Academy of Sciences
28	Hussain et al	2021	Circular economy approach to recycling technologies of post-consumer textile waste in Estonia: a review	Journal of Enterprise Information Management
29	Jing et al	2021	Towards the Circular Economy: Converting Aromatic Plastic Waste Back to Arenes over a Ru/Nb2O5 Catalyst	Angewandte Chemie
30	Kazancoglu et al	2020	A conceptual framework for barriers of circular supply chains for sustainability in the textile industry	Sustainable Development
31	Keller and Bette	2020	Shaping digital sustainable development in chemical companies	Journal of Business Chemistry
32	Kouhizadeh, Zhu and Sarkis	2020	Blockchain and the circular economy: potential tensions and critical reflections from practice	Production Planning & Control
33	Laibach, Börner and Bröring	2019	Exploring the future of the bioeconomy: An expert-based scoping study examining key enabling technology fields with potential to foster the transition toward a bio-based economy	Technology in Society
34	Luo et al	2016	Value-added processing of crude glycerol into chemicals and polymers	Bioresource Technology
35	Massaya et al	2019	Conceptualization of a spent coffee grounds biorefinery: A review of existing valorisation approaches	Food and Bioproducts Processing
36	Milovanovic et al	2018	Supercritical CO2 impregnation of PLA/PCL films with natural substances for bacterial growth control in food packaging	Food Research International
37	Morone, Tartiu and Falcone	2015	Assessing the potential of biowaste for bioplastics production through social network analysis	Journal of Cleaner Production
38	Mukherjee et al	2019	Use of bio-based polymers in agricultural exclusion nets: A perspective	Biosystems Engineering
39	Nilsen-Nygaard et al	2021	Current status of biobased and biodegradable food packaging materials: Impact on food quality and effect of innovative processing technologies	Comprehensive Reviews in Food Science and Food Safety
40	Nižetić et al	2019	Smart technologies for promotion of energy efficiency, utilization of sustainable resources and waste management	Journal of Cleaner Production
41	Pagoropoulos, Pigosso and McAloone	2017	The Emergent Role of Digital Technologies in the Circular Economy: A Review	Procedia
42	Pinales-Márquez et al	2021	Circular bioeconomy and integrated biorefinery in the production of xylooligosaccharides from lignocellulosic biomass: A review	Industrial Crops & Products
43	Puyol et al	2017	Resource recovery from wastewater by biological technologies: Opportunities, challenges, and prospects	Frontiers in Microbiology
44	Ranta, Aarikka-Stenroos and Väisänen	2021	Digital technologies catalyzing business model innovation for circular economy—Multiple case study	Resources, Conservation & Recycling
45	Saberi et al	2019	Blockchain technology and its relationships to sustainable supply chain management	International Journal of Production and Research
46	Sahajwalla and Hossain	2020	The science of microrecycling: a review of selective synpaper of materials from electronic waste	Materials Today Sustainability
47	Satchatippavarn et al	2016	Urban biorefinery for waste processing	Chemical Engineering Research and Design
48	Sovacool et al	2021	Decarbonizing the food and beverages industry: A critical and systematic review of developments, sociotechnical systems and policy options	Renewable and Sustainable Energy Reviews
49	Tian et al	2019	Organic waste to biohydrogen: A critical review from technological development and environmental impact analysis perspective	Applied Energy
50	Unruh	2018	Circular Economy, 3D Printing, and the Biosphere Rules	California Management Review
51	Vollmer et al	2020	Beyond Mechanical Recycling: Giving New Life to Plastic Waste	Angewandte Chemie
52	Vrchota et al	2020	Sustainability outcomes of green processes in relation to industry 4.0 in manufacturing: Systematic review	Sustainability
53	Wu and Montalvo	2021	Repurposing waste plastics into cleaner asphalt pavement materials: A critical literature review	Journal of Cleaner Production
54	Zeiss et al	2021	Mobilising information systems scholarship for a circular economy: Review, synpaper, and directions for future research	Information Systems Journal
55	Žnidaršič-Plazl	2021	Let the Biocatalyst Flow	Acta Chimica Slovenica

## Footnotes

1 To process and analyse the data considering the different perspectives put forward in the literature, the concept is divided in two. Whereas the analysis in this action area focuses on the feasibility of production of renewable biological resources, the technologies that enable the transformation of waste into value-added products are discussed in the ‘Loop’ action area.
